# Feasibility Study on Additive Manufacturing of Ferritic Steels to Meet Mechanical Properties of Safety Relevant Forged Parts

**DOI:** 10.3390/ma15010383

**Published:** 2022-01-05

**Authors:** Linda Mally, Martin Werz, Stefan Weihe

**Affiliations:** 1 Institute for Materials Testing, Materials Science and Strength of Materials (IMWF), University of Stuttgart, Pfaffenwaldring 32, 70569 Stuttgart, Germany; stefan.weihe@mpa.uni-stuttgart.de; 2 Materials Testing Institute, University of Stuttgart, Pfaffenwaldring 32, 70569 Stuttgart, Germany; martin.werz@mpa.uni-stuttgart.de

**Keywords:** ferritic steel, heat treatment, SLM, additive manufacturing, nuclear application

## Abstract

Additive manufacturing processes such as selective laser melting are rapidly gaining a foothold in safety-relevant areas of application such as powerplants or nuclear facilities. Special requirements apply to these applications. A certain material behavior must be guaranteed and the material must be approved for these applications. One of the biggest challenges here is the transfer of these already approved materials from conventional manufacturing processes to additive manufacturing. Ferritic steels that have been processed conventionally by forging, welding, casting, and bending are widely used in safety-relevant applications such as reactor pressure vessels, steam generators, valves, and piping. However, the use of ferritic steels for AM has been relatively little explored. In search of new materials for the SLM process, it is assumed that materials with good weldability are also additively processible. Therefore, the processability with SLM, the process behavior, and the achievable material properties of the weldable ferritic material 22NiMoCr3-7, which is currently used in nuclear facilities, are investigated. The material properties achieved in the SLM are compared with the conventionally forged material as it is used in state-of-the-art pressure water reactors. This study shows that the ferritic-bainitic steel 22NiMoCr3-7 is suitable for processing with SLM. Suitable process parameters were found with which density values > 99% were achieved. For the comparison of the two materials in this study, the microstructure, hardness values, and tensile strength were compared. By means of a specially adapted heat treatment method, the material properties of the printed material could be approximated to those of the original block material. In particular, the cooling medium/cooling method was adapted and the cooling rate reduced. The targeted ferritic-bainitic microstructure was achieved by this heat treatment. The main difference found between the two materials relates to the grain sizes present. For the forged material, the grain size distribution varies between very fine and slightly coarse grains. The grain size distribution in the printed material is more uniform and the grains are smaller overall. In general, it was difficult and only minimal possible to induce grain growth. As a result, the hardness values of the printed material are also slightly higher. The tensile strength could be approximated to that of the reference material up to 60 MPa. The approximation of the mechanical-technological properties is therefore deemed to be adequate.

## 1. Introduction

Additive manufacturing (AM) processes have evolved in a short time from rapid prototyping—the production of prototypes and sample components, to industrially applicable components and structures [1]. The national and international effort to bring these additively manufactured components into use in safety-relevant areas such as power plants or nuclear facilities is great [2]. The production processes developed for the second generation of nuclear power plants during the first peak of nuclear technology (Generation II Peak) have become obsolete due to dismantling of existing plants, restructuring of production facilities (scrapping of machinery, takeover, etc.), and loss of knowledge (retirement of employees), and can no longer be applied without considerable effort and expense [3]. Currently, we are seeing a second peak (Generation III Peak) in the nuclear power sector, with some countries like Germany dealing with the dismantling of nuclear facilities, while in other areas of the world new plant systems are being built (Gen. III), developed (Gen. IV) and commissioned. Therefore, new advanced production processes and manufacturing techniques such as additive manufacturing are being relied upon for future spare parts, the development, and new construction of power plants worldwide [3]. According to the German Gesellschaft für Reaktorsicherheit (GRS) [4], the trend in the development of new reactors and reactor concepts is moving towards Small Modular Reactors (SMR). As a result, component sizes are likely to decrease in the future and new manufacturing methods such as additive manufacturing could be applied. Among the most important advanced manufacturing technologies in this regard, based on industry interest, is selective laser beam melting (SLM/L-PBF), according to the United States Nuclear Regulatory Commission (USNRC) [2]. Currently, the SLM process is mainly used for the production of small to medium-sized components. In the field of nuclear technology, additively manufactured valves and other non-safety-critical small to medium-sized components are already being used. The industry’s interest in also being able to manufacture large components with this process is significant and is leading to the development of ever-larger machines. For example, the SLM^®^800 developed by SLM Solutions already has a build envelope with dimensions of 500 × 290 × 850 mm [5]. Another example of the rapid growth of the available built envelope dimensions is the SLM laboratory system developed by the Aachen University of Applied Sciences and the Fraunhofer Institute for Laser Technology ILT. Their system has an effectively usable built envelope of 1000 × 800 × 500 mm, which is considerably larger than other previous commercial SLM systems [6]. This trend makes the use of additively manufactured components in the reactor sector feasible and could lead to the manufacturing of large components.

The principle of the laser and powder bed-based manufacturing process (SLM/L-PBF) is a layer-by-layer build-up, in which a component is created using selective melting of metal powder with a laser beam [1]. The resulting component properties are determined by a variety of influencing factors, such as machine size, powder properties, process parameters, resulting imperfections, etc. In total, there are more than 130 quality-deciding influencing factors [7]. In addition, each material exhibits different behavior in the additive manufacturing process and must therefore be investigated separately. The most commonly used materials for L-PBF are AlSi10Mg [8,9], Ti6Al4V [10,11], Inconel [12] or 316L [13,14]. These have been studied in more detail in the past, so optimized process parameter sets for these materials are available in the literature. However, the parameter sets presented in the published studies are often incomplete or are not transferable to other manufacturing equipment because of the selected performance settings. In addition, it has been found that even the same parameter sets on identical production equipment in different sites can lead to different results, for example in terms of porosity and mechanical strength, so that the parameters must be adapted separately for each machine [15].

This study focuses on the ferritic reactor steel 22NiMoCr3-7 as this material was used almost exclusively in reactor pressure vessel construction in the Federal Republic of Germany [16]. The optimized reactor steel 22NiMoCr3-7 was derived from the American material ASTM A 508 Cl.2 (20NiMoCr3-6). The ASTM A 508 Cl.2 low-alloy (higher-strength) steel was selected for use in German nuclear power plants on account of its mechanical properties (especially toughness), which are adequate even for large wall thicknesses, its good through-temperability, its low tendency to radiation embrittlement, and it is at this time considered good weldability. The optimized reactor steel 22NiMoCr3-7 had also been considered well suited for welded joints. Later, however, this steel proved to be sensitive to embrittlement and cracking in weld-affected zones under certain circumstances [16]. Experiments were conducted at the Materials Testing Institute (MPA) when this welding issue was discovered analyzing the reason and influencing factors for relaxation embrittlement and crack formation in 22NiMoCr3-7. These experiments confirmed the only cause for the development of relaxation embrittlement and crack formations to be overheating of the material with coarse grain formation and if the initially dissolved metallic carbides are precipitated at the grain boundaries because of rapid cooling followed by subsequent heat treatment. These precipitates are responsible for additional hardening of the material and reduce both its creep tendency and its ductility in the grain boundary zones. The residual stresses generated during welding are relieved during conventional two-stage stress relief annealing by plastic creep deformation. In damaged grain boundary regions, however, the creep and strain capacity is quickly exhausted, and inter-granular cracking may occur. It was found out that the main decisive influencing factors are, in addition to the acting stresses, the chemical steel composition, as well as the kinetics of the precipitation processes in the heat-affected zone (HAZ), which depend on the temperature-time processes during welding and the subsequent heat treatment [16].

The problems with the welding safety of the steel 22NiMoCr3-7 and its tendency to undercladding and side cracks led to a discussion of whether 20MnMoNi5-5 might not be more suitable. However, the discussion about the competing reactor steels eased after a strong narrowing of the chemical analysis limits, preferably for molybdenum and trace elements accompanying the steel, which was recommended after the investigations of the MPA Stuttgart, so that the steel 22NiMoCr3-7 could be substantially improved in its properties. The limit values that should not be exceeded are shown in Table 1. According to the Reactor Safety Committee, the modified 22NiMoCr3-7 is a desirable alternative to the steel 20MnMoNi5-5 [16]. Because of its still important role as one of the main materials for reactor pressure vessels erected in the Federal Republic of Germany, it is therefore of interest for future research endeavors with regard to spare parts production using additive manufacturing processes. Possible problems arising with regard to weldability and thus processability using additive manufacturing were investigated in this study. The material behavior of the conventionally manufactured 22NiMoCr3-7 has already been extensively investigated in past studies and research projects at the MPA of the University of Stuttgart. The material properties, as well as the material and failure behavior of the conventionally manufactured material, with which the obtained results of this study are compared, were for example elaborated by Seebich [17].

In literature, there are only a few published studies on the subject of processing ferritic materials using L-PBF [19,20,21,22,23,24]. To date, there are no empirical values or published parameter windows for the processability of 22NiMoCr3-7 using the L-PBF method. The parameter sets given in the literature cannot be applied unchanged for the reasons already mentioned, but serve as a data basis for future process parameter variations. Moreover, the use of ferritic powder materials such as 22NiMoCr3-7 in the L-PBF process makes the manufacturing process and the creation of the desired microstructure and the associated material properties even more complex. Due to the higher cooling rates > $10^{6\;\circ }$C/s [25] during the manufacturing process, subsequent heat treatment is required to obtain the desired ferritic-bainitic microstructure instead of the expected martensitic structure present after printing. Initial studies on microstructure adjustment with subsequent heat treatment for ferritic materials exist [19] and are used for comparison purposes.

However, to establish additive manufactured components (AM components) and structures in safety-critical and certification-relevant application areas, the process-related material properties and the resulting material behavior must be fundamentally understood and mastered. The challenges that have to be overcome in the future include anisotropic microstructures, non-uniform stress distributions and pore formations as well as many other effects. Only when additive manufacturing processes have been sufficiently validated can the economic and technical advantages of this technology be used without hesitation in safety-critical areas such as nuclear power plants.

## 2. Materials and Methods

### 2.1. Material

**Figure 1:**
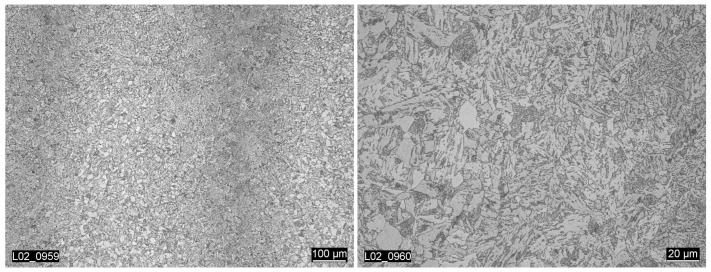
Textures and lines of the forged block material [17].

The material block of 22NiMoCr3-7 used in this study was taken from the upper forged core ring of the reactor pressure vessel originally planned for the nuclear Biblis-C plant. The material is a ferritic-bainitic fine-grained structural steel. The microstructure of the block material displays the lines and textures typical for a forged material. The mentioned lines can be seen in the vertical color differences visible in the left image in Figure 1. For this material, the heat treatment takes place after the forging process, which makes the texture slightly less obvious. The grain size varies from very fine to slightly coarse [17], as can be seen in the right image in Figure 1.

The hardness resulted in an average value of 189 HV 30. Further investigations of the material in previous projects show that the material has very homogeneous and isotropic material properties [17]. The results of the performed tensile test are shown in Table 2.

The steel powder was produced by atomizing the 22NiMoCr3-7 block material provided by the Materials Testing Institute (MPA) Stuttgart at Höganäs. The influence of the particle size on the flowability of metal powder material was taken into account when selecting the particle size range. In this respect, it was considered that fine powder particles tend to form agglomerates and thus impair flowability. However, fine particles can fill the voids of the coarser particles and help to increase the density of the powder layer, which leads to a better process result [26]. Based on experience and typical industry specifications for steel powder for L-PBF production, 15 µm was chosen as the lower limit. The upper limit was set at 45 µm.

The specification for the chemical composition of 22NiMoCr3-7 including limit values and an overview of the chemical compositions of the block and powder material is shown in Table 3.

The chemical composition of the original block material is compared with the composition of the resulting target grain to guarantee the correct alloy composition even after pulverization. In addition, information on welding behavior, microstructure development and the effect of a subsequent heat treatment strategy can be derived from the listed composition. The chemical composition of the block material was determined using a Quantovac analysis at MPA Stuttgart [17], while the powder was analyzed at the manufacturer’s site. Since losses of certain alloying elements can occur during the production process, for example due to burn-off, the chemical composition was also checked after the production of the specimens by means of Spark Optical Emission Spectrometry (F-OES). The results for the chemical composition of the as-printed material are discussed in Section 3.1.

### 2.2. Manufacturing

At present, there is almost no information to be found on the additive processing of reactor steel, especially on 22NiMoCr3-7. Therefore, no parameter sets for the processing of 22NiMoCr3-7 by means of SLM are available. There is also little information on the general processing of ferrites or high-temperature steels. One study dealing with a material similar in area of application, namely a strong and ductile Reduced Activation Ferritic/Martensitic (RAFM) steel employed for fusion reactors [23], is used as a starting point for the parameter variation of this study. Jiang et al. provides a complete defined process parameter window with parameters adaptable to the equipment available for this study. The laser power varied between 160 W and 320 W at scanning speeds of 400 to 1200 mm/s, while keeping the layer thickness constant at 30 µm and a hatch spacing of 85 µm. The parameter combinations of 200 W and scanning speed of 800 mm/s, as well as laser power of 320 W and scanning speed of 600 mm/s proved to be the most suitable for the RAFM material [23]. To verify whether this parameter window deviates strongly from other materials frequently used in additive manufacturing, the process parameters of the austenitic steel 1.4404 were also considered. 1.4404 is one of the most common steels processed by SLM because the material is very inexpensive and has very good processing properties. For this reason, there are already process parameter sets available. Laser powers between 150 W and 250 W and scanning speeds of 600–950 mm/s have proven suitable for this material on the Aconity Mini System used in this study. It was found that even for different materials with different properties, the parameter windows overlap to a large extent. Therefore, parameters from this range or slightly above or below were selected for the parameter determination of this study. The process parameter variations carried out are listed in Table 4.

**Figure 2:**
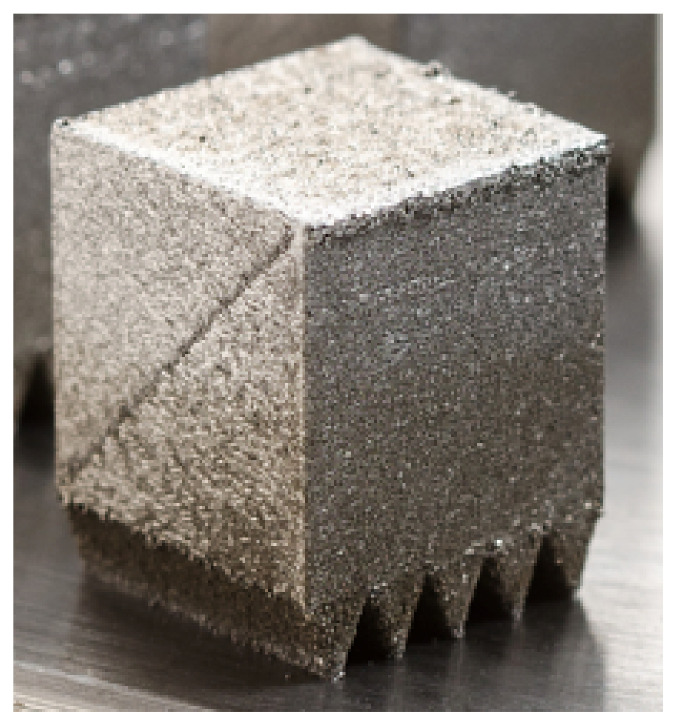
Specimen cube with a wedge-shaped support structure.

The processability of 22NiMoCr3-7 was investigated on cube-shaped specimens with different process parameter variations using an Aconity Mini system (Aconity3D). The printed cubes were designed with wedge-shaped solid support structures, as seen in Figure 2, to ensure a secure bond to the build plate. An edge length of 10 mm was chosen for the cubes. The layer thickness was set constant at 30 µm for all cubes. The hatch spacing and laser spot diameter were not changed during the parameter variation. A simple hatching scan strategy was chosen for the entire cross-section, which was rotated 90° for each subsequent layer. The contour was exposed after filling. The manufacturing was performed under an argon atmosphere at an oxygen content below 100 ppm. The build plate was initially not preheated to investigate the influence of the high cooling rate during the printing process on the resulting microstructure.

### 2.3. Density Measurement

The relative density of the additively manufactured cube specimens achieved with the selected process parameters was used as a determining criterion for assessing the suitability of the parameters used. The density was determined according to Archimedes’ principle (ASTM B962-17) and additionally by microscopic image analysis of a cut surface of the cube specimens. For this purpose, one cube per parameter set was cut in the center parallel to the direction of build-up. Subsequently, the cut surfaces of each specimen were polished to visualize the pore size and distribution.

### 2.4. Heat Treatment and Hardness Measurement

**Figure 3:**
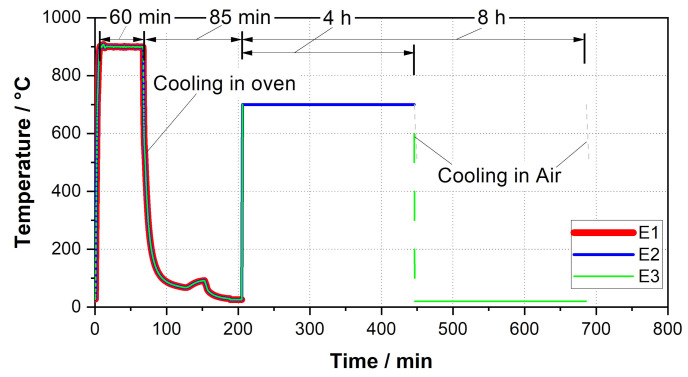
Controlled heat treatment for E1–E3.

To approximate the mechanical properties of the additively manufactured material to those of the original forging block material, various heat treatment strategies were investigated. The performed heat treatment variations are listed in Table 5. The temperature profile for the specimens cooled in the oven in a controlled environment is shown in Figure 3.

Following the heat treatment, sections were again prepared to analyze the resulting microstructure. In addition, Vickers hardness tests were carried out in accordance with DIN EN ISO 6507-1.

### 2.5. Tensile Testing

**Figure 4:**
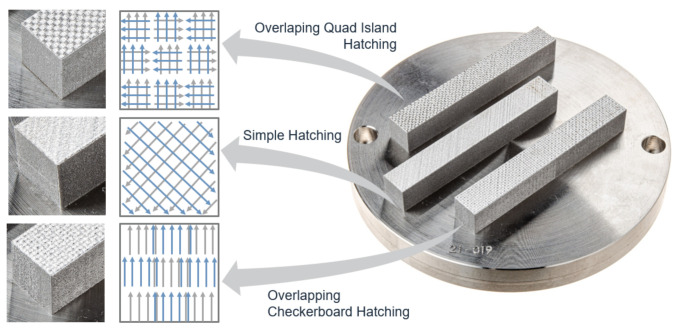
Specimens with different scan strategies.

For further material characterization, tensile specimen blanks (TSB) with dimensions 12 mm × 12 mm × 86 mm were manufactured in horizontal orientation (0°). The parameter set PV1 (laser power = 250 W, scan speed = 700 mm/s) was used for the production. The layer thickness was left at 30 µm. Fabrication also took place in an argon atmosphere. For the fabrication of these specimens, to minimize residual stresses, the build platform was heated to 150 °C. In addition to the tensile test specimens printed with the simple hatching scan strategy (designation according to pre-process software Netfabb Autodesk) three additional scanning strategies (see Figure 4) were tested to estimate their effect on the resulting material properties.

**Figure 5:**
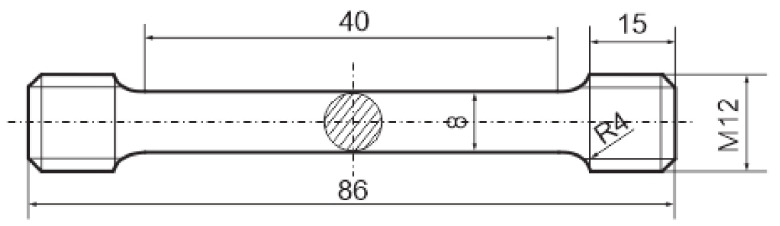
Tensile test specimen B 8 X 40 DIN 50125.

The tensile specimen blanks were heat-treated before machining the target contour by lathing according to B 8 X 40 DIN 50125 (see Figure 5) to prevent decarbonization of the layers near the surface. The tensile tests were performed in accordance with DIN EN ISO 6892-1:2020-06 on a Zwick Roell 100 kN tensile testing machine Typ BZ1-MMZ100.ZW02.

## 3. Experimental Results

### 3.1. Material

The results of the chemical analysis of the printed material show that the amount of alloying elements present is still within specification, see Table 2. However, significant deviations from the powder analysis are noticeable in some cases. The manganese content has decreased because of vaporization during by the manufacturing process. An increase in chromium, nickel and copper was detected compared to the results of the atomized powder material. As the measured values of these elements in the printed material are very similar to those of the block material, it is assumed that the chemical analysis of the powder material is inaccurate due to the different material state (powder versus solid).

The fact that material burns off during the process is confirmed by the fact that the filters in the area of the process gas circulation are completely black and clogged after the process. The results of the chemical analysis of the material deposited on the filters are still pending and will provide further information on the loss of alloying elements.

### 3.2. Density Measurement and Parameter Evaluation

The results of the density test are shown below in Table 6. There were significant differences in the relative density for the different process parameter sets used. Parameter set PV1 showed the highest density values. Moreover, the specimens showed a good surface quality. PV2 and PV3 showed a considerably worse surface quality compared to PV1.

**Figure 6:**
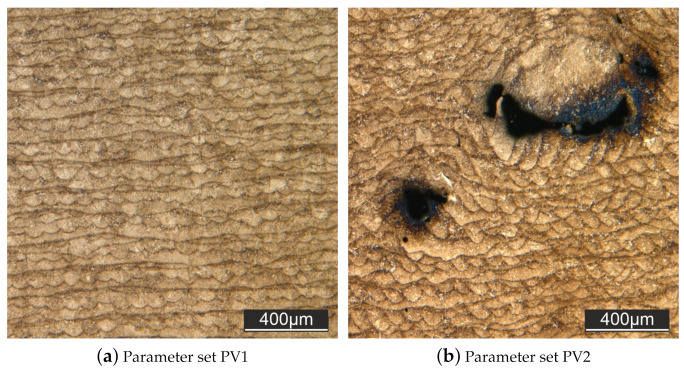
Micrograph without defects (**a**) and with defects (**b**).

The measurements according to the Archimedean principle could be quantitatively confirmed by light microscopic images of the micrographs. The micrographs of parameter set 2 (PV2) show a relatively large number of flaws and attachment defects (see Figure 6a), whereas PV1 (see Figure 6b) and PV3 hardly show any discontinuities.

Except for the area of influence of the flaws, all samples show a uniform layer thickness. The tested parameter variations PV4 and PV5 are not suitable for the processing of the investigated material. In the case of PV4 unwanted material accumulations on the surface occurred during the manufacturing process and in the case of PV5, only a poor bond to the building board was achieved. Parameter set 1 (PV1) was therefore used for all further tests.

### 3.3. Heat Treatment and Hardness Measurement

**Figure 7:**
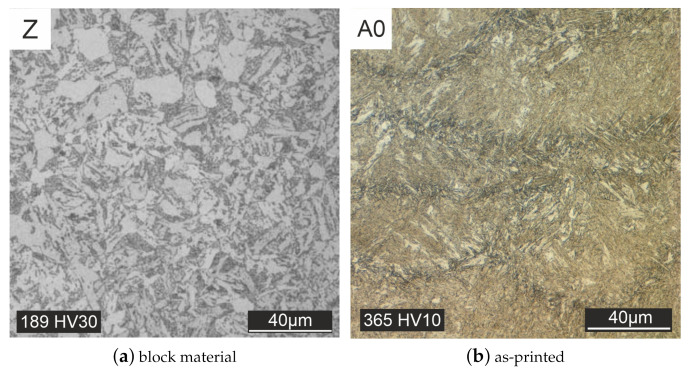
Microstructure forged block material (**a**) and as-printed material (**b**).

**Figure 8:**
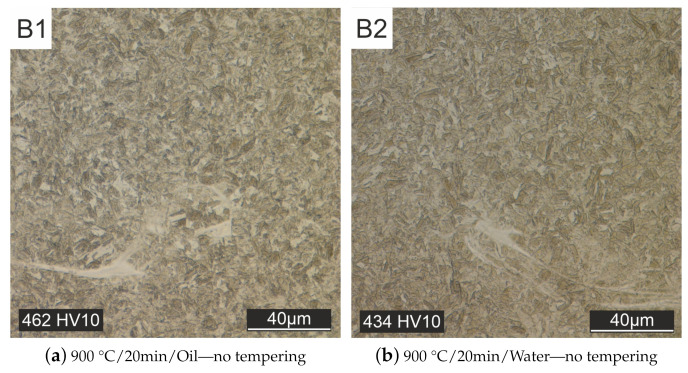
Heat treated but not tempered specimens, quenched with oil (**a**) or water (**b**).

**Figure 9:**
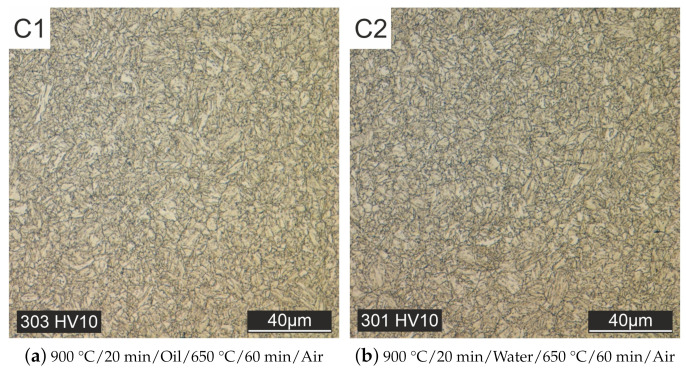
Heat treated and tempered specimens, quenched with oil (**a**) or water (**b**).

**Figure 10:**
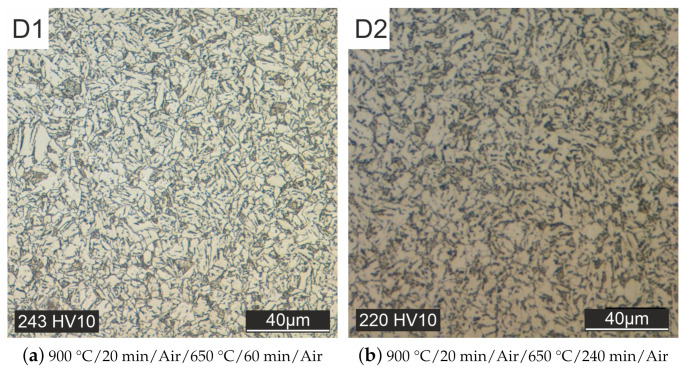
Heat treated with different tempering times and cooling in air.

**Figure 11:**
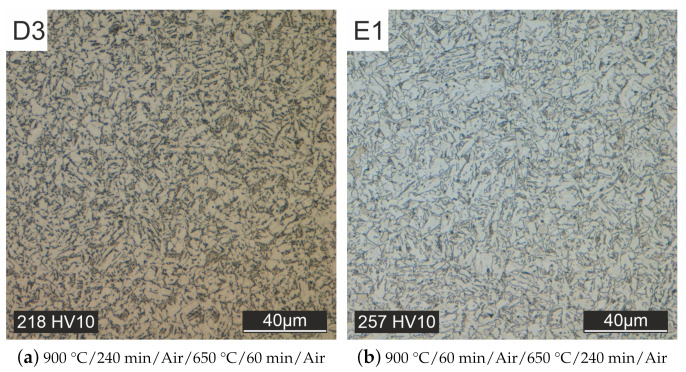
Optimized cooling in air (**a**) and first attempt at controlled cooling in the oven (**b**).

**Figure 12:**
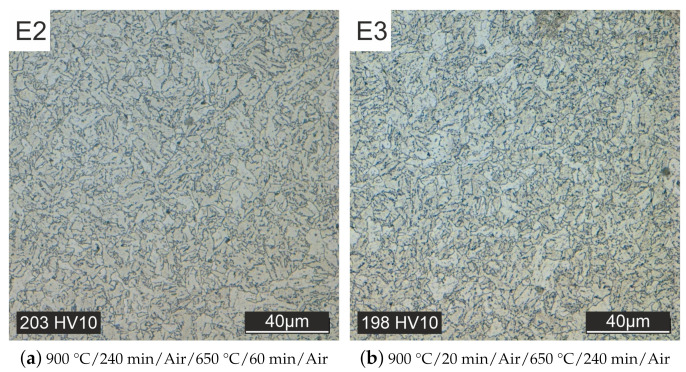
Different controlled cooling strategies in the oven.

The aim of the heat treatment was to approximate the microstructure of the as-printed material, as well as the mechanical properties to that of the forged material. The target microstructure of the forged block material (Z) is displayed in Figure 7a and shows a ferritic-bainitic microstructure with fine to slightly coarse grain size. In Figure 7b the as-printed state (A0), the individual melting traces of the manufacturing process are clearly visible. The results of the different heat treatment strategies are shown in Figure 8, Figure 9, Figure 10, Figure 11 and Figure 12.

In contrast to the as-printed material, the melt traces are no longer visible in the cross-section of all heat-treated specimens. Homogenization of the microstructure takes place during the heat treatment. Initially, heat treatments were carried out without subsequent tempering (Figure 8). Quenching after austenitizing took place in oil Figure 8a or water Figure 8b. With these heat treatment strategies, only a martensitic microstructure could be produced (see Figure 8).

The bright streaks in the microstructure image probably originate from particle splashes that were ejected from the melt pool and landed back on the specimen surface, resulting in a different cooling rate than the rest of the sample. These streaks are no longer visible in the micrographs after other heat treatment strategies with subsequent tempering.

An additional tempering process at 650 °C for 60 min followed by cooling in air lowers the hardness value, but only a or fine-grained martensitic microstructure could be achieved, see Figure 9a,b. The additional tempering process at 650 °C for 60 min followed by cooling in air lowers the hardness value, but the microstructure is still martensitic. The results show that quenching in oil and water is too fast so that the bainite region cannot be reached. Therefore, for the subsequent heat treatment tests, cooling in the air was carried out after austenitizing at 900 °C and then tempering at 650 °C for 60 min.

The slower cooling process in air allowed to reach the bainitic region, see Figure 10a. However, a very fine structure with small grains is still present here.

At this point, a significant increase in grain size is estimated to be only possible through overheating. This heat treatment, however, deviates from the specifications of the material manufacturer. Therefore, the grain size was supposed to be increased by a longer tempering time (specimen D2, see Figure 10b) or austenitizing time (specimen D3, see Figure 11a). This goal could not be achieved, only the hardness could be lowered further.

Heat treatment with a controllable cooling process for both austenitizing and tempering allows further reduction of hardness and slight grain growth (specimen E2 and E3, see Figure 12).

For subsequent component tests, the duration of austenitizing and tempering must be determined as a function of the dimensions. The heat treatment of the component tests can therefore deviate from the heat treatment strategy determined here.

### 3.4. Tensile Performance

**Figure 13:**
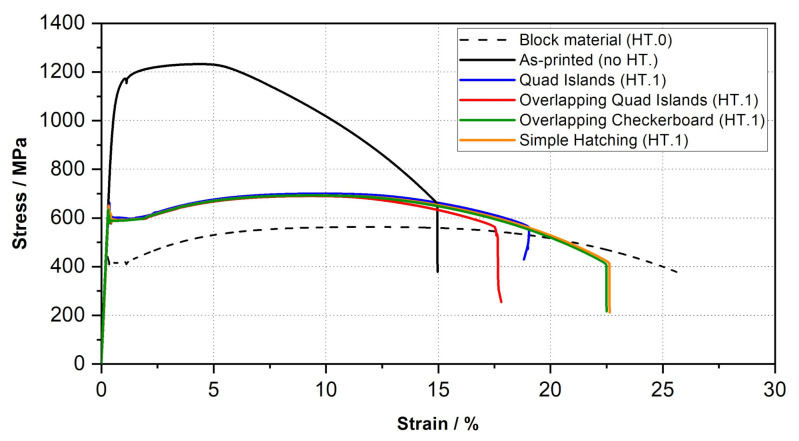
Stress-Strain curves - 22NiMoCr3-7 at RT for specimens with different scan strategies.

The results of the tensile tests showed a substantial difference between the block material and the as-printed material (see Figure 13), which was to be expected because of the difference in the presented microstructure and the corresponding mechanical properties. The dashed black curve (block material) corresponds to the conventionally produced forged material and the black curve to the as-printed condition. The as-printed specimen was produced using the simple hatching scanning strategy, parameter set PV1, and was not heat treated. The forged block material possesses a tensile strength of UTS = 563 MPa and an elongation at fracture of A = 26%. In comparison the as-printed material has a significantly higher tensile strength with a value of UTS = 1230 MPa and an elongation at fracture of A = 15%. All other specimens shown in Figure 13 were manufactured using the same parameter set (PV1) as the as-printed material and in addition, subject to the D3 heat treatment strategy (900 °C/240 min/Air/650 °C/60 min/Air) available at the time of testing. Further on the D3 heat treatment is also referred to as HT.1 because it was considered the first successful heat treatment strategy. Applying heat treatment D3 significantly reduced the tensile strength. The tensile strength of the specimens could thereby be approximated to a difference of 140 MPa to the forged block material. Moreover, the plots of the stress-strain curves of the specimens with different scan strategies confirm a correlation of the applied scanning strategy with the ductility of the additively manufactured material.

**Figure 14:**
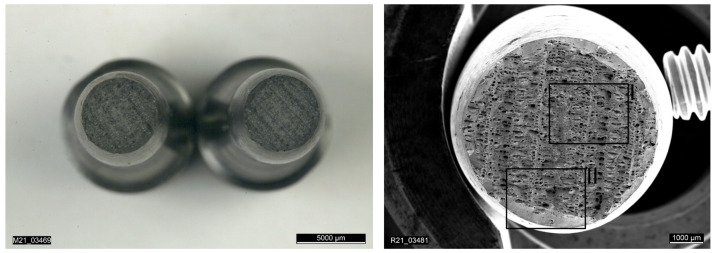
Overview of the fracture surface of a specimen printed with the quad island scan strategy.

**Figure 15:**
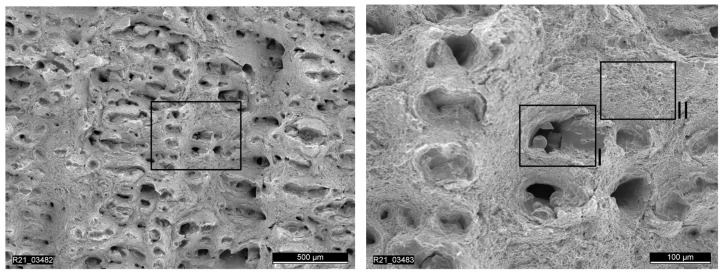
Detailed image showing lack of fusion and uniform material sections on fracture surface using quad island scan strategy.

The first three tensile test specimens were manufactured using the quad island strategy. The results of the tensile tests showed strong scattering with regard to the achieved elongation at fracture, as can be seen in Table 7. Analysis of the fracture surface of specimen RTS-1 under the electron microscope reveals lack of fusion between the quad islands (see Figure 14 and Figure 15) resulting from the selected scan strategy. The result from the specimen RTS-1 is plotted in dark blue in Figure 13.

To further increase the elongation at fracture an overlap of individual islands within the quad island scan strategy was tested. For this purpose, the hatch spacing was reduced, while the laser spot diameter remained the same, to achieve a larger overlap of the individual fusion tracks. The stress-strain curve in Figure 14 shows, however, that this adjustment, plotted in red, does not lead to an improvement for the elongation at break. In contrast, the scanning strategies of the specimens RTS-5 (simple-hatching, orange) and RTS-6 (overlapping checkerboard, dark green) visibly improve the toughness.

**Figure 16:**
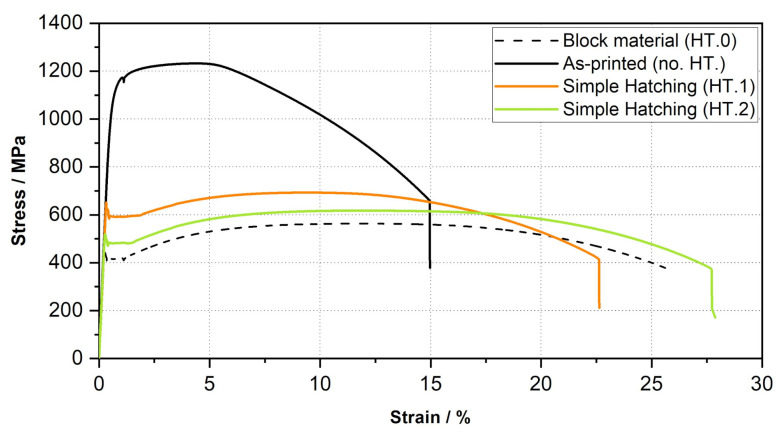
Stress-Strain curves—22NiMoCr3-7 at RT for specimens with different heat treatment strategies.

Continued optimization of the D3 heat treatment (HT.1) lead to the E3 strategy (HT.2) with even better results concerning the tensile performance (see the green curve in Figure 16).

An evaluation of the results of the new tensile tests shows that the elongation at break could be approximated to within 4% of that of the forged block material using the HT.2 method. The goal of achieving the same tensile strength of the bulk material could not be fully achieved due to the grain refinement caused by L-PBF. There is a remaining deviation of 60 MPa.

## 4. Conclusions

In this study, the ferritic reactor steel 22NiMoCr3-7 was successfully processed additively using the L-PBF process. The focus of the investigation was on defining a suitable process parameter window for selective laser melting of the selected material. The laser power, scan speed, and scan strategy were varied to develop a suitable process window. A set of process parameters (PV1) was developed that achieved a relative density of over 99%. In addition, different scanning strategies were tested and their effect on the resulting tensile strength and toughness was investigated. The scan strategy named simple hatching showed the best results. The parameter set PV1 and the named scan strategy were used for subsequent sample fabrication and investigation of various post-treatment heat strategies. The additively manufactured samples with no heat treatment showed insufficient ductile material behavior in the as-printed state, as well as a martensitic microstructure. The goal to approximate the microstructure and the mechanical-technological properties of the forged block material was first successful with the D1 heat treatment. The desired ferritic-bainitic microstructure was obtained. Even the hardness values (218 HV10) were in a similar range to those of the forged material (189 HV30). Further optimization of the heat treatment strategy adding a controlled cooling process in an oven achieved even better results concerning the grain size of the microstructure as well as the hardness values (198 HV10). The development of the E3 heat treatment concludes the search for a suitable heat-treatment process. With this heat treatment, it was possible to achieve the original microstructure of the forged block material as well as its mechanical-technological properties. Based on these investigations, tensile specimens were manufactured and tested. The results showed very good agreement between the additively manufactured and forged specimens. In addition, further experiments on notch tensile, shear tensile, and flat tensile specimens with cracks are in preparation and should show that the material behavior with varying stress multiaxiality is similar for additively manufactured and forged material.

## Figures and Tables

**Table 1:** Crack initiation after exceeding two or more of these values [18].

Limit Contents in Weight Percent					
**Mo**	**P**	**S**	**Cu**	**Sn**	**N**
0.62	0.008	0.008	0.12	0.011	0.013

**Table 2:** Strength and deformation characteristics from tensile test [17].

L${}_{0}$	UTS	UYP	A	A${}_{g}$	Z
[mm]	[MPa]	[MPa]	[%]	[%]	[%]
50	584	445	23.0	10.7	68

**Table 3:** Specification [27] and overview of chemical composition for 22NiMoCr3-7.

	C	Si	Mn	Cr	Mo	Ni	P	S	Cu	Sn	Al	V	Ta	Co	As
Spec min.	0.17	0.10	0.50	0.25	0.50	0.60	-	-	-	-	-	-	-	-	-
Spec max.	0.25	0.35	1.00	0.50	0.75	1.00	0.012	0.015	0.1	-	0.05	0.05	0.03	0.03	-
block	0.21	0.20	0.88	0.4	0.53	0.83	0.006	0.002	0.039	0.007	0.016	0.007	<0.003	0.011	0.005
powder	0.19	0.22	0.93	0.28	0.51	0.67	0.006	0.004	0.007	0.02	0.02	<0.01	<0.01	0.01	-
as-printed	0.197	0.21	0.79	0.43	0.547	0.93	0.007	0.004	0.042	0.007	0.014	0.007	0.004	0.013	0.006

**Table 4:** Parameter variation.

Parameter Set	Laser Power [W]	Scan Speed [mm/s]
PV1	250	700
PV2	200	800
PV3	150	800
PV4	300	1000
PV5	250	1525

**Table 5:** Heat treatment strategies.

Strategy	Austenitization	Austenitization	Cooling	Tempering	Tempering
	Temperature	Time	Medium	Temperature	Time
	[${}^{\circ }$C]	[min]		[${}^{\circ }$C]	[min]
Z	900		water	650	450
A0	-	-	-	-	-
B1/B2	900	20	oil/water	-	-
C1/C2	900	20	oil/water	650	60
D1	900	20	air	650	60
D2	900	20	air	650	240
D3	900	240	air	650	60
E1	900	60	oven (100 °C)	-	-
E2	900	60	oven (100 °C)	700	240
E3	900	60	oven (100 °C)	700	480

**Table 6:** Density measurement according to the Archimdedean principle.

Specimen	Laser Power [W]	Scan Speed [mm/s]	Density [%]
PV1-1	250	700	99.10
PV1-2	250	700	98.07
PV1-3	250	700	99.60
PV2-1	200	800	90.74
PV2-2	200	800	95.17
PV2-3	200	800	91.57
PV3-1	150	800	90.74
PV3-2	150	800	99.58
PV3-3	150	800	95.93

**Table 7:** Strength and deformation characteristics.

Specimen	Diameter	L_0_	UTS	UYP	A	A_g_	Z
	[mm]	[mm]	[MPa]	[MPa]	[%]	[%]	[%]
Block material	no information	50	584	445	23.0	10.7	68
As-printed	7.96	40	1230	-	15.0	-	68
RTS-2	8.00	40	700	665	19.0	-	51
RTS-3	8.01	40	703	636	18.0	-	48
RTS-4	7.98	40	690	646	17.5	9.6	55
RTS-5	7.98	40	693	651	22.0	9.7	71
RTS-6	7.96	40	693	635	22.0	9.8	71
RTS-7	8.00	40	623	492	24	12.0	57
RTS-8	8.00	40	618	510	23	11.8	57
RTS-9	8.00	40	618	519	28	12.2	72
RTS-10	8.00	40	618	517	27	12.0	70
RTS-11	8.00	40	616	510	26	11.8	73
RTS-12	8.00	40	622	469	27	12.2	72

## Data Availability

Not applicable.

## Abbreviations

The following abbreviations are used in this manuscript:

AM	Additive manufacturing
SLM	Selective Laser Melting
L-PBF	Laser Powder Bed Fusion
GRS	Gesellschaft für Reaktorsicherheit
SMR	Small Modular Reactors
USNRC	United States Nuclear Regulatory Commission
MPA	Materials Testing Institute
HAZ	heat-affected zone
L0	Measuring length
UTS	Ultimate tensile strength
UYP	Upper yield point
A	Elongation at fracture
Ag	Uniform elongation
UTS	Ultimate tensile strength
Z	Necking
F-OES	Spark Optical Emission Spectrometry
RAFM	Reduced Activation Ferritic/Martensitic
TSB	tensile specimen blank
